# Chronic Q Fever in Alberta: A Case of* Coxiella burnetii* Mycotic Aneurysm and Concomitant Vertebral Osteomyelitis

**DOI:** 10.1155/2016/7456157

**Published:** 2016-05-11

**Authors:** William Stokes, Jack Janvier, Stephen Vaughan

**Affiliations:** ^1^University of Calgary, Calgary, AB, Canada; ^2^Division of Infectious Diseases, South Health Campus, 4th Floor, Room 480073, 4448 Front Street SE, Calgary, AB, Canada T3M 1M4

## Abstract

Chronic Q fever is a potentially life-threatening infection from the intracellular, Gram-negative* Coxiella burnetii*. It presents most commonly as endocarditis or vascular infection in people with underlying cardiac or vascular disease. We discuss a case of a 67-year-old male with* Coxiella burnetii* vascular infection of a perirenal abdominal aortic graft. The patient had a history of an abdominal aortic aneurysm (AAA) repair 5 years earlier. He presented with a 12 × 6 × 8 cm perirenal pseudoaneurysm and concomitant L1, L2, and L3 vertebral body discitis. He underwent an open repair which revealed a grossly infected graft perioperatively. Q fever serology revealed phase I serological IgG titer of 1 : 2048 and phase II 1 : 1024 consistent with chronic Q fever. Polymerase chain reaction (PCR) on infected vascular tissue was positive for* C. burnetii*. The patient was started on doxycycline and hydroxychloroquine with good clinical response and decreasing serological titers. Recognizing chronic Q fever is a difficult task as symptoms are nonspecific, exposure risk is difficult to ascertain, and diagnosis is hidden from conventional microbiological investigations. Its recognition, however, is critical as* C. burnetii* is inherently resistant to standard empiric therapies used in cardiovascular infections.

## 1. Introduction

*Coxiella burnetii* is an obligate intracellular, Gram-negative bacteria that replicates inside host cell phagosomes. Its animal reservoir is vast but farm animals such as sheep, cattle, and goats predominate. Inhalation of infected fomites causes acute infection, known as acute Q fever, which generally presents as an asymptomatic or mild, spontaneously resolving, illness [1].

A major complication in 2–6% of acute Q fever cases is progression, within months to years, into chronic Q fever [2–4]. It presents most commonly as endocarditis or vascular infection in people with underlying cardiac or vascular disease [5].

Recognizing chronic Q fever is a difficult task as symptoms are generally nonspecific, exposure risk is difficult to ascertain, and diagnosis is hidden from conventional microbiological investigations. Its recognition, however, is critical as* C. burnetii* is inherently resistant to standard empiric therapies used in cardiovascular infections [6].

We present a case of a 67-year-old male with* Coxiella burnetii* vascular infection of a perirenal abdominal aortic graft. This is the first documented case of* C. burnetii* vascular infection in Canada and the second documented case of chronic Q fever in Alberta [7].

## 2. Case

A 67-year-old male, originally from Southeastern Europe, was admitted to hospital in 2011 after a 12 × 6 × 8 cm perirenal abdominal aortic aneurysm (AAA) was found on ultrasound during workup for acute lower back and abdominal pain. The patient had a previous AAA open repair 5 years earlier with no complications. He also had chronic renal disease secondary to hypertension (baseline serum creatinine 140–180 *μ*mol/L), past history of heavy smoking (quit 5 years earlier, total pack years unknown), and chronic mechanical low back pain managed medically for the last four years. He had immigrated to Canada from Southeastern Europe in 2009 and was living in Southern Alberta. He had previously worked as a mechanical technician. He denied any recent or past exposures to farm animals or unpasteurized dairy products. The patient denied fatigue, weight loss, night sweats, fevers, or chills. Vital signs were normal and the patient was afebrile. Physical exam demonstrated poor distal pulses and a palpable aorta with the remainder of the exam being normal. Laboratory workup revealed a longstanding normocytic anemia (Hgb 125 g/L) and elevated creatinine (174 *μ*mol/L). Liver enzymes and function were within normal limits. C-reactive protein was elevated at 156.8 mg/L.

**Figure 1:**
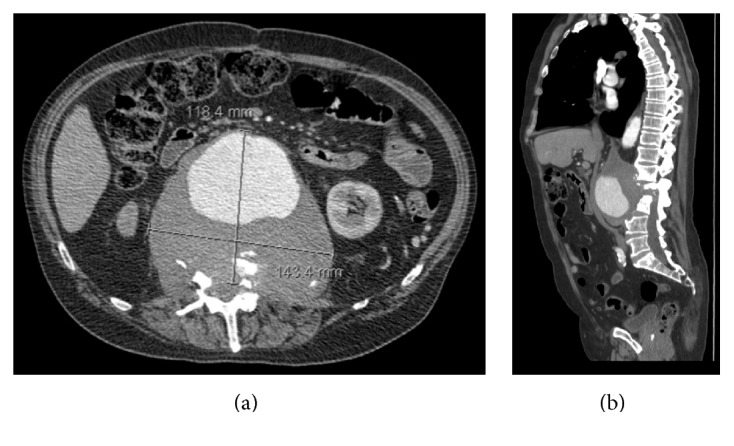
Single images from the CT angiogram scan of the perirenal saccular abdominal pseudoaneurysm with adjacent discitis. (a) Axial image of the pseudoaneurysm 11.8 × 14.3 cm in dimensions. (b) Sagittal image of the pseudoaneurysm with concomitant L1–L3 discitis.

Computed tomography (CT) angiogram demonstrated an 11.8 × 14.3 cm perirenal saccular abdominal pseudoaneurysm arising from the posterior aspect of the proximal anastomosis of his abdominal aortic graft repair (Figure 1(a)). The sac extended posteriorly with extensive destruction of L1, L2, and L3 vertebral bodies (Figure 1(b)). Follow-up magnetic resonance imaging revealed L2-L3 disk space destruction that caused a moderate degree of spinal stenosis and severe left neural foraminal compromise at the L2-L3 level. It was noted that a smaller (11.4 × 7.3 cm) aortic mass with L1–L3 destruction was observed on noncontrast CT 1 year prior to his presentation, though no interventions were taken at that time. Based on the CT and MRI findings, it was felt that the pseudoaneurysm was most likely a complication from the patient's previous AAA repair (anastomotic pseudoaneurysm). Infection was not suspected.

The patient underwent an open, redo repair of his abdominal aortic aneurysm and bilateral renal artery reconstruction. The operation revealed a contained AAA rupture with large amounts of purulence and massive erosion of the patient's adjacent lumbar spine with exposure to anterior elements but no visible dura. Pathology demonstrated normal appearing atherosclerotic plaque with no signs of infection or granulomas.

One day after operation the patient developed respiratory distress as a result of renal failure from prerenal acute tubular necrosis. He was transferred to the intensive care unit for mechanical ventilation. During his stay he had recurrent fevers (Tmax 39.2) despite prolonged (approximately 1 month) treatment of piperacillin/tazobactam and vancomycin for presumed mycotic aneurysm. Aerobic and anaerobic bacterial and fungal cultures of blood and infected vascular tissue were negative, including cultures specific for* Mycoplasma*,* Brucella*, and* Mycobacteria*. Screening for human immunodeficiency virus, hepatitis B, hepatitis C, and syphilis was negative. After 1 month, Q fever serology results returned positive with phase I IgG serological titer of 1 : 2048 and phase II 1 : 1024, consistent with a diagnosis of chronic Q fever.* Coxiella burnetii* IgM was negative. PCR on the vascular tissue, completed postmortem, was positive for* C. burnetii*.

The patient was treated for chronic Q fever with doxycycline 100 mg twice daily and hydroxychloroquine 200 mg daily, with plan for reassessment once fully recuperated. Response to treatment was verified by repeat Q fever serology 20 months later revealing decreased titers of 1 : 512 (phase I) and 1 : 256 (phase II).

Though the patient had multiple complications during his 3-month hospital stay, the patient was successfully discharged to another centre for ongoing rehabilitation and shortly thereafter returned home with his family. He died 18 months later (22 months since admission) from hypoxia and multiorgan failure secondary to an acute pulmonary embolism (PE) confirmed on CT angiography. The PE was not thought to be related to Q fever since the patient had previously improved from his hospital stay and had decreasing phase I and II serological titers.

## 3. Discussion

*Coxiella burnetii* has an affinity for abnormal cardiac and vascular tissue as well as implanted grafts and artificial valves. Abdominal aortic aneurysms and AAA grafts are the most common sites of endovascular infection with* C. burnetii*. Other risk factors include older age, male gender, immunodeficiency, smoking, and chronic renal disease [5, 8, 9].

Chronic Q fever symptoms are nonspecific. In a case series of 58* C. burnetii* endovascular infections, presenting symptoms included fever (70%), abdominal pain (62%), lumbar pain (12%), weight loss (43%), and fatigue (24%) [10]. Concomitant spondylitis was present in 15% of cases and was not always associated with lumbar pain.

Given its nonspecific presentation, vascular chronic Q fever is a clinically difficult diagnosis to secure as it is not seen on Gram stains or routine cultures. Instead, a combination of PCR, serology, and imaging is needed. Culture is rarely performed as it is a highly infectious organism that can place laboratory personnel at risk of infection. The two phases of chronic Q fever are best detected serologically with indirect immunofluorescence assay (IFA). While enzyme-linked immunosorbent assay (ELISA) and complement fixation assay (CFA) are other methods used in detecting Q fever, especially acute, they are less sensitive than IFA for chronic Q fever [11]. IFA, from Focus Diagnostics, Inc., is 97.8% sensitive for chronic Q fever when phase I IgG serological titer is >1,024 [5]. However, the positive predictive value is only 62.2% and 66.7% for phase I titers of 1 : 1,024 and 1 : 2,048, respectively, reinforcing the importance of using PCR and imaging, in addition to serology, for diagnosis [5].

Two diagnostic guidelines specific for* Coxiella burnetii* vascular infections exist [12, 13]. Our patient meets the requirements for* Coxiella burnetii* vascular infection according to both criteria based on PCR, serology, imaging, and clinical findings.

Chronic Q fever is difficult to treat given its inherent resistance to many antibiotics. It is treated for a minimum of 18 months months with hydroxychloroquine and doxycycline based on a prospective study from France that demonstrated no relapses of Q fever endocarditis with the combination drug after 18 months [14]. Vascular infections are treated similarly despite the lack of studies. Therefore, it is important to monitor the serological titer response of treatment and, as in our patient, consider prolonging treatment duration on a case-by-case basis.

There has been one documented case of chronic Q fever in Alberta, a 31-year-old male, with congenital valvular disease, diagnosed with Q fever endocarditis after workup for congestive heart failure and fever [7]. As far as this writer can tell there are no published cases of vascular Q fever infection in Alberta or Canada to date.

We hope this case report addresses the importance for* Coxiella burnetii* testing in culture-negative mycotic aneurysms. Any patient with a suspected culture-negative vascular infection should undergo serological testing for* C. burnetii*, with the diagnosis made in accordance with serological titer, PCR on blood and/or vascular tissue, relevant imaging, and clinical context. Diagnosis is critical as* C. burnetii* is intrinsically resistant to standard mycotic aneurysm empiric therapies. In addition, our case is the first documented chronic Q fever vascular infection within Canada and the second chronic Q fever case in Alberta. With increasing globalization, an aging population, and better diagnostic methods,* Coxiella burnetii* may play a larger role in vascular infections within Canada and Alberta in the future.
